# Strangulated Adult Bochdalek Hernia With Extensive Small Bowel Necrosis and Septic Shock

**DOI:** 10.7759/cureus.115081

**Published:** 2026-08-24

**Authors:** Karol Polett Nuñez Gamarra, Daniel Medina-Neira, Martin Eugenio Manzaneda Peralta

**Affiliations:** 1 Faculty of Human Medicine, Universidad Católica de Santa María, Arequipa, PER; 2 Department of Internal Medicine, St. Bernards Medical Center, Jonesboro, USA; 3 Department of Surgery, Hospital Nacional Carlos Alberto Seguín Escobedo, Arequipa, PER

**Keywords:** adult congenital diaphragmatic hernia, adult diaphragmatic hernia, congenital diaphragmatic hernia, intestinal resection, laparotomy repair, septic shock, small bowel ischemia, small intestinal resection

## Abstract

Bochdalek diaphragmatic hernias are congenital diaphragmatic defects through which abdominal viscera herniate into the thoracic cavity and are typically diagnosed in newborns and infants. Adult presentations are uncommon and may present with heterogeneous manifestations, ranging from asymptomatic to life-threatening complications. A 72-year-old male with a history of urinary retention presented with acute lower abdominal pain and vomiting. Initial physical examination and laboratory studies suggested a urinary tract infection. Despite adequate treatment, the patient’s condition deteriorated, and progressive hemodynamic instability led to the diagnosis of septic shock secondary to intestinal obstruction. Chest X-ray incidentally revealed intrathoracic bowel loops, raising suspicion for a diaphragmatic hernia, while computed tomography confirmed the Bochdalek hernia with strangulated bowel. Emergency laparoscopic exploration identified a 3 x 3 cm diaphragmatic defect with herniated small bowel. Due to irreducible herniation and dense adhesions, the procedure was converted to laparotomy. The defect was enlarged to facilitate reduction, followed by resection of 1.2 m of necrotic small bowel, primary diaphragmatic repair, and bowel anastomosis. The patient recovered and remained recurrence-free after four years. This case underscores that adult diaphragmatic hernias may initially present with nonspecific symptoms in patients with comorbidities that suggest more common diagnoses, sometimes leading to premature diagnostic closure. Prompt reassessment and broad differential diagnosis for patients with clinical deterioration and unclear etiology are key to timely recognition of complicated adult diaphragmatic hernias. Early surgical management can then ensure favorable outcomes even in severe, life-threatening complications.

## Introduction

Non-hiatal diaphragmatic hernias (DHs) are rare defects of the diaphragm that allow abdominal organs to migrate into the thoracic cavity through an abnormal opening other than the esophageal hiatus. They are broadly categorized as traumatic or congenital. Congenital diaphragmatic hernias (CDHs) are rare, with a prevalence ranging from 1.7 to 5.7 per 10,000 births [1]; Bochdalek hernias account for 90% of cases, while Morgagni hernias comprise 5% [2]. Bochdalek hernias result from failure of closure of the posterolateral pleuroperitoneal membrane, occurring predominantly on the left side, whereas Morgagni hernias result from anterior retrosternal defects, favoring the right side. CDH in adults is exceedingly rare, with an estimated prevalence ranging from 0.17% [3] to 6% [4], frequently discovered as incidental findings on routine CT imaging. Clinical presentation in this group is highly heterogeneous, ranging from asymptomatic to acute complications, including obstruction, strangulation, volvulus, or incarceration, which can lead to ischemia, perforation, or hemodynamic instability [1,4-6]. Adult Bochdalek hernia may be overlooked until complications develop, as concurrent comorbidities often mask symptoms related to diaphragmatic defects, leading to diagnostic delays.

We report a rare case of a late-presenting adult Bochdalek DH. We highlight the diagnostic challenges of overlapping comorbidities, the critical role of targeted imaging, and the successful open repair of a primary diaphragmatic defect. Despite severe systemic compromise, primary repair combined with small bowel resection and side-to-side anastomosis resulted in an excellent clinical outcome.

## Case presentation

A 72-year-old male with a history of prostatic enlargement and an indwelling urinary catheter presented with three hours of acute colicky lower abdominal pain, nausea, and vomiting. His only relevant prior surgical history was a bilateral inguinal hernioplasty performed 20 years earlier.

On admission, blood pressure (BP) was 130/75 mmHg, heart rate (HR) was 85 beats per minute, respiratory rate was 19 breaths per minute, oxygen saturation was 95% on room air, and the patient was afebrile. Physical examination revealed lower abdominal and bilateral costovertebral angle tenderness. Laboratory studies showed mild leukocytosis, elevated C-reactive protein, impaired renal function, and abnormal urinalysis (Table 1). These findings suggested a urinary tract infection (UTI) with obstructive uropathy. Intravenous (IV) ceftriaxone (2 g every 24 hours) was started. By the third hospital day, the patient developed worsening abdominal pain, constipation, and hemodynamic instability (BP of 70/40 mmHg and HR of 110 beats/min). Laboratory evaluation showed lactic acidosis, leukocytosis, and worsening renal function, findings compatible with septic shock (Table 1). The report of an upright abdominal X-ray indicated dilated bowel loops and multiple air-fluid levels, compatible with intestinal obstruction. Immediate resuscitation was initiated. A chest X-ray report obtained to confirm central venous catheter position also described cephalad displacement of the nasogastric tube and intrathoracic bowel loops, raising suspicion for DH.

**Table 1:** Laboratory findings during hospitalization. WBC, white blood cells; CRP, C-reactive protein; BUN, blood urea nitrogen; HPF, high-power field.

Laboratory test	Reference range	Admission	Hospital day 3 (Surgery day)
WBC (x10^3^/µL)	4-10	11.12	26.56
Segmented neutrophils (%)	40-70	92	82
Band neutrophils (%)	0-5	3	12
CRP (mg/dL)	<0.5	2.5	34.1
Creatinine (mg/dL)	0.7-1.3	2.16	4.14
BUN (mg/dL)	7-20	57	80.2
Lactate (mmol/L)	0.5-1.6	-	6.3
Urine leukocytes (cells/HPF)	0-5	>100	-
Urine erythrocytes (cells/HPF)	0-2	Abundant	-
Urine bacteria	Negative	+++	-

**Figure 1:**
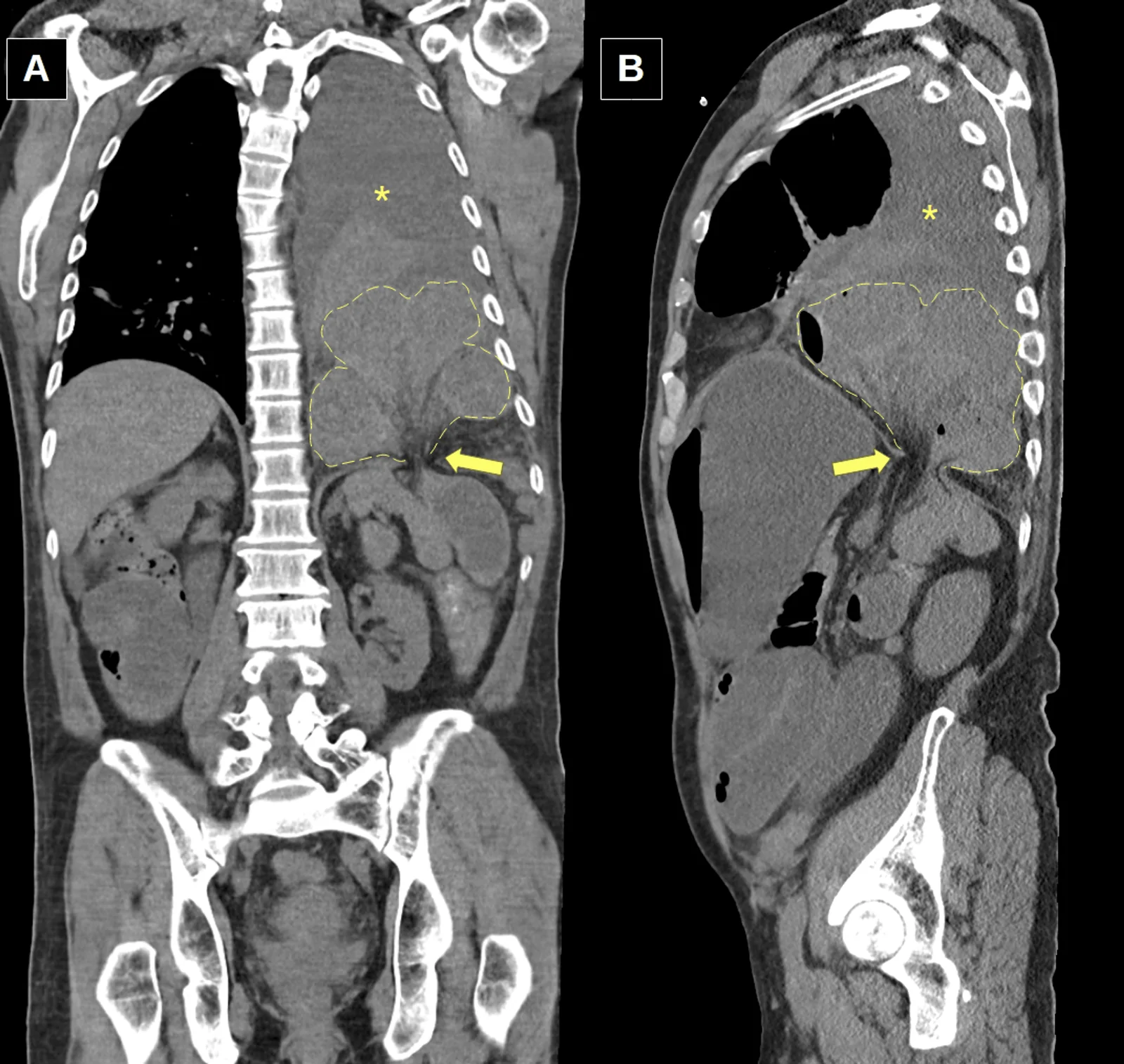
Multiplanar CT images demonstrating herniation of the small bowel through a left-sided diaphragmatic defect. (A) Coronal view and (B) sagittal view. Images show herniated bowel loops (dashed lines) traversing the defect. The asterisk (*) marks indeterminate structures, possibly herniated omentum versus collapsed lung, not definitively distinguishable on these views.

A computed tomography (CT) scan confirmed a left posterior diaphragm defect with herniated bowel and thoracoabdominal complications (Figure 1). The diagnosis of strangulated DH in a patient in shock was made, and IV meropenem (1 g every eight hours) and metronidazole (500 mg every eight hours) were initiated, followed by emergency surgical intervention on the same day of the diagnosis (third day of hospitalization).

Laparoscopic exploration revealed distended small bowel loops herniating through a 3 × 3 cm left hemidiaphragm defect. The herniated content was irreducible, so the procedure was converted to open laparotomy. The defect was enlarged to approximately 8 cm to facilitate mobilization. Intraoperative findings included hemorrhagic pleural fluid, dense pleuro-omental adhesions, necrotic omentum, and a 1.2 m segment of necrotic small bowel located 2.2 m distal to the ligament of Treitz and 2 m proximal to the ileocecal valve. Following adhesiolysis and reduction of herniated contents, a chest tube was placed toward the left lung base under direct visualization. The diaphragmatic defect was repaired primarily in two layers using interrupted U-shaped sutures, followed by a continuous invaginating layer, achieving a tension-free closure. Partial omentectomy of the necrotic segment was performed; the 1.2-meter ischemic bowel segment was resected using a linear stapling device, and intestinal continuity was restored via a side-to-side anastomosis. This was followed by abdominal irrigation and placement of a Penrose drain extending into the pelvic cavity. The abdominal wall was closed in standard fashion.

In the immediate postoperative period, the patient underwent a single hemodialysis session for severe metabolic acidosis, with improvement in renal function and no need for further renal replacement therapy. Mechanical ventilation and vasopressor support were required for two days. IV meropenem and vancomycin were continued for two weeks postoperatively, and an oral diet was resumed on postoperative day 10. Following an uneventful remaining hospital course, the patient was discharged on postoperative day 16 and remains asymptomatic without recurrence at four years.

## Discussion

We present the case of an elderly patient with a left DH complicated by strangulation and extensive intestinal necrosis, presenting acutely with septic shock secondary to complicated bowel obstruction, an uncommon and potentially fatal presentation. The absence of a trauma history and the left posterolateral defect location were consistent with a late-presenting Bochdalek hernia.

According to the literature, a small CDH may occasionally remain asymptomatic until adulthood, as gradual herniation permits progressive visceral accommodation [3,7-9]. However, it can debut when acute incarceration precipitates bowel obstruction and vascular compromise [1]. This heterogeneous presentation, combined with atypical symptom localization and confounding comorbidities, frequently delays recognition.

Our patient’s lower abdominal pain and laboratory findings consistent with UTI, in the context of known prostatic disease, initially supported a reasonable urologic diagnosis, which may have contributed to two well-recognized cognitive biases in diagnostic reasoning: anchoring and premature diagnostic closure. Anchoring occurs when initial information disproportionately influences subsequent clinical judgment, whereas premature closure refers to accepting a plausible diagnosis before it has been sufficiently verified, limiting consideration of alternative explanations [10]. In this case, the laboratory profile compatible with UTI and pre-existing prostatic disease may have reinforced the initial diagnosis, while the nonspecific nature of the abdominal pain created diagnostic overlap, as the symptoms could be attributed to UTI but also represented an early manifestation of intestinal compromise secondary to complicated DH. Such biases may be particularly likely when a common diagnosis provides a coherent explanation for the initial presentation, potentially delaying recognition of less common conditions such as complicated DH in adulthood, a pattern previously described [11-13]. In our case, the diagnosis was ultimately delayed until progression of abdominal symptoms despite adequate treatment, prompting reassessment and evaluation of the differential diagnosis. Finally, imaging played a key role in identifying the etiology, as a chest X-ray incidentally revealed intrathoracic bowel loops, and CT confirmed the diagnosis, establishing both the anatomical location of the diaphragmatic defect and the presence of bowel strangulation [14].

Our case differs from previously reported cases of strangulated DH in several respects. The patient lacked the upper abdominal or thoracic pain typically described in this condition [13,15], an atypical presentation that may delay diagnosis. Also, the extent of intestinal necrosis (1.2 m of small bowel) was substantially greater than in prior reports, including one case with only 20 cm of necrotic bowel that nonetheless progressed to perforation, a complicated postoperative course, and death [16]. Despite the larger extent of ischemia in our patient, the outcome was favorable. Finally, systemic compromise was notably more severe [12,17-19], characterized by septic shock, a rarely reported manifestation of this condition, and hemothorax, both of which resolved without long-term consequences.

In our patient, surgical intervention was indicated due to progressive hemodynamic deterioration and complicated bowel involvement [14,20]. Laparoscopy was chosen to confirm the diagnosis and assess bowel viability. However, due to dense adhesions and the inability to reduce the incarcerated viscera, conversion to laparotomy was required. In this setting, conversion should not be considered a failure but rather a strategy to achieve safe reduction and definitive treatment. Intentional enlargement of the diaphragmatic defect was key to enabling atraumatic visceral reduction and preventing further injury to already compromised intestinal loops. Hemorrhagic pleural fluid and dense pleuroparietal adhesions within the left hemithoracic cavity supported placement of a chest tube for postoperative drainage and lung re-expansion. The diaphragmatic defect, although enlarged, allowed primary closure without tension. Mesh reinforcement was considered but ultimately deferred due to the increased risk of infection in the setting of intestinal necrosis and potential contamination.

## Conclusions

Our case illustrates that adult DH may remain clinically silent for years before presenting acutely with life-threatening complications, such as intestinal obstruction and strangulation. Coexisting comorbidities and nonspecific initial symptoms can orient the diagnosis toward more common conditions, significantly delaying recognition of the underlying cause. In this patient, reassessment prompted by clinical deterioration, together with an incidental chest X-ray and subsequent advanced imaging, was essential for establishing the correct diagnosis. Despite the severity of presentation, timely source control through bowel resection and diaphragmatic repair achieved durable long-term outcomes, demonstrated by the absence of recurrence or procedure-related complications after four years of follow-up.

DH should not be excluded from the differential diagnosis of acute abdomen in adults. Avoiding cognitive biases, such as premature diagnostic closure and anchoring, is essential, as its presentation frequently overlaps with those of more common conditions. An initially plausible diagnosis should not preclude reassessment when the clinical course deviates from the expected trajectory, particularly in patients with confounding comorbidities. Maintaining a high index of suspicion, obtaining early imaging, and pursuing prompt surgical management are key to timely intervention. These measures may help prevent progression to bowel ischemia, septic shock, and extensive bowel resection, ultimately improving outcomes even in patients presenting with severe complications.
